# Toxin profiles and antimicrobial resistance patterns among toxigenic clinical isolates of *Clostridioides (Clostridium) difficile*

**DOI:** 10.22038/ijbms.2019.35223.8390

**Published:** 2019-07

**Authors:** Hamid Heidari, Hadi Sedigh Ebrahim-Saraie, Ali Amanati, Mohammad Motamedifar, Nahal Hadi, Abdollah Bazargani

**Affiliations:** 1 Department of Bacteriology and Virology, School of Medicine, Shiraz University of Medical Sciences, Shiraz, Iran; 2 Professor Alborzi Clinical Microbiology Research Center, Shiraz University of Medical Sciences, Shiraz, Iran; 3 Shiraz HIV/AIDS Research Center, Institute of Health, Shiraz University of Medical Sciences, Shiraz, Iran; 4 Bioinformatics and Computational Biology Research Center, Shiraz University of Medical Sciences, Shiraz, Iran

**Keywords:** Antibiotic resistance, CDT, Clostridioides (Clostridium) – difficile, C. difficile infection, Toxins

## Abstract

**Objective(s):**

*Clostridioides (Clostridium)* *difficile* infection as a healthcare-associated infection can cause life-threatening infectious diarrhea in hospitalized patients. The aim of this study was to investigate the toxin profiles and antimicrobial resistance patterns of *C. difficile* isolates obtained from hospitalized patients in Shiraz, Iran.

**Materials and Methods:**

This study was performed on 45 toxigenic *C. difficile* isolates. Determination of toxin profiles was done using polymerase chain reaction method. Antimicrobial susceptibility to vancomycin, metronidazole, clindamycin, tetracycline, moxifloxacin, and chloramphenicol was determined by the agar dilution method. The genes encoding antibiotic resistance were detected by the standard procedures.

**Results:**

The most frequent toxin profile was *tcdA+*, *tcdB+*, *cdtAˉ*, *cdtBˉ *(82.2%), and only one isolate harboured all toxin associated genes (*tcdA*+, *tcdB+*, *cdtA+*, *cdtB+*) (2.2%). The genes encoding CDT (binary toxin) were also found in six (13.3%) isolates. Resistance to tetracycline, clindamycin and moxifloxacin was observed in 66.7%, 60% and 42.2% of the isolates, respectively. None of the strains showed resistance to other antibiotics. The distribution of the *ermB* gene (the gene encoding resistance to clindamycin) was 57.8% and the *tetM* and *tetW* genes (the genes encoding resistance to tetracycline) were found in 62.2% and 13.3% of the isolates, respectively. The substitutions Thr82 to Ile in GyrA and Asp426 to Asn in GyrB were seen in moxifloxacin resistant isolates.

**Conclusion:**

Our data contributes to the present understanding of virulence and resistance traits amongst the isolates. Infection control strategies should be implemented carefully in order to curb the dissemination of *C. difficile* strains in hospital.

## Introduction

*Clostridioides difficile* (formerly *Clostridium difficile*) is a Gram-positive rod-shaped, spore-forming, strictly anaerobic bacterium and the causative agent of *C. difficile* infection (CDI) (1). CDI, as a serious healthcare-associated infection, can cause life-threatening infectious diarrhea in hospitalized patients (2). Disruption of the intestinal microbiome induced by antibiotics is the major risk factor for the development of CDI (3).

Toxins secreted by the toxigenic strains are responsible for the occurrence of the disease. *C. difficile* toxins A and B are the main virulence factors of the pathogens, encoded by *tcdA *and* tcdB* genes, respectively. They are located on the pathogenicity locus (PaLoc). Toxin A as an enterotoxin and Toxin B with cytotoxic activity alter the actin cytoskeleton and cause cell rounding, disruption of tight junctions, and intestinal function failure (3-5).

Besides toxins A and B, some of *C. difficile* strains express a binary toxin (CDT). This additional virulence factor belongs to the ADP-ribosyltransferase family and consists of enzymatic (CDTa) and binding components (CDTb) (3-5). The *cdtA* and *cdtB* genes encode the CDT toxin that induces actin depolymerization in the cytoskeleton, leading to an increase in the bacterial adherence to colonic epithelium and severity of infection (5, 6).

The risk of CDI is increased if *C. difficile* is resistant to the antibiotics used. Although *C. difficile *is usually susceptible to metronidazole and vancomycin (the first line of antibiotics used for the treatment of CDI), resistance or decreased susceptibility has been reported in the literature (7, 8). 

Mechanisms of resistance to the members of macrolide–lincosamide–streptogramin B (MLS_B_) group include ribosomal modification, efflux pumps, and drug inactivation. Resistance to MLS_B_ in *C. difficile* is mainly associated with the *erm* genes (especially *ermB*) located on a mobile genetic element (7, 9, 10). Other probable mechanisms such as efflux could have caused this type of resistance (7).

In *C. difficile*, resistance mechanisms to other antimicrobial agents such as tetracyclines, fluoroquinolones and chloramphenicol were also characterized previously. The *tet* and *catD* genes confer tetracyclines and chloramphenicol resistance, respectively. Different mutations in *gyrA* and *gyrB* are also associated with quinolones resistance (7, 10, 11).

To the best of our knowledge, there are few data regarding CDI in Iran. Therefore, the aim of the present study was to evaluate the toxin profiles and antimicrobial resistance patterns of *C. difficile* isolates obtained from hospitalized patients with CDI in Shiraz, southwestern Iran.

## Materials and Methods

***Bacterial isolates***

This study was performed on 45 toxigenic *C. difficile* isolates obtained from fecal specimens of patients with antibiotic-associated diarrhea, hospitalized in Nemazee Hospital (The main hospital affiliated to Shiraz University of Medical Sciences) and Amir Oncology Hospital from October 2017 to June 2018 (12). Only one isolate per patient was included. This study was approved by the Ethics Committee of Shiraz University of Medical Sciences (Register code: IR.SUMS.REC.1397.114).

According to the World Health Organization (WHO), diarrhea was defined as more than 3 loose and watery stools during a 24 hr period or fewer hrs (13). Also, based on the European Centre for Disease Prevention and Control (ECDC), CDI was defined as a patient with diarrhea whose stool takes the container shape, and is positive for toxigenic (toxin A and/or toxin B) *C*. *difficile* without any other etiology (14). Identification of the isolates was performed based on Gram staining, odor and colony characteristics on cycloserine-cefoxitin fructose agar (CCFA) plate. The *tpi* housekeeping gene (triose phosphate isomerase) was targeted by polymerase chain reaction (PCR), using specific primers for molecular confirmation (Table 1) (15). The PCR protocol consisted of a pre-denaturation step at 95 ^°^C for 5 min, followed by 30 cycles of 60 sec at 95 ^°^C, 45 sec at 53 ^°^C and 50 sec at 72 ^°^C. A final extension step was performed at 72^°^C for 5 min.

***Determination of toxin profile***

**Figure 1:**
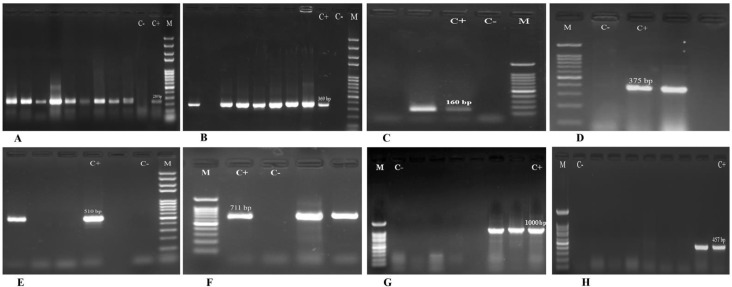
Agarose gel electrophoresis of PCR products for *tpi* (A), *tcdA* (B), *tcdB* (C), *cdtA* (D), *cdtB* (E), *ermB* (F), *tetM* (G) and *tetW* (H) genes. Lane M: 100 bp DNA size marker; Lane C-: negative control; Lane C+: positive control

Genomic DNA was extracted from freshly grown colonies, using the commercial DNA extraction kits (GeneAll, Korea) according to the manufacturer’s instructions. PCR was carried out for detection of the genes encoding toxin A and toxin B (*tcdA* and *tcdB*) and binary toxin CDT (*cdtA* and *cdtB*) by specific primers (Table 1) (15, 16). The PCR reactions consisted of a pre-denaturation step at 95 ^°^C for 5 min, followed by 30 cycles of 60 sec at 95 ^°^C, 45 sec at 51 ^°^C (for *tcdA*), 50 ^°^C (for *tcdB*), 53 ^°^C (for *cdtA* and *cdtB*), and 50 sec at 72 ^°^C. A final extension step was performed at 72 ^°^C for 5 min. The PCR products were separated by electrophoresis in 1% agarose gels with 1 X TAE (Tris/Acetate/EDTA) buffer, stained with safe stain load dye (CinnaGen Co., Iran) and visualized under ultraviolet (UV) illumination (Figure 1).

***Antimicrobial susceptibility testing***

The minimum inhibitory concentrations (MICs) of six antibiotics including vancomycin, metronidazole, clindamycin, tetracycline, moxifloxacin, and chloramphenicol were determined by the agar dilution method according to the Clinical and Laboratory Standards Institute (CLSI) (17). Brucella agar supplemented with hemin (5 µg/ml), vitamin K1 (10 µg/ml), and 5% sheep blood was used for the tests. *C. difficile* ATCC 700057 was used as a control strain for the susceptibility tests. Antibiotic resistance and susceptibility were determined using the breakpoints defined by the CLSI (for metronidazole, clindamycin, tetracycline, moxifloxacin, and chloramphenicol) and the European Committee on Antimicrobial Susceptibility Testing (EUCAST) (for vancomycin) (18).

***Detection of antimicrobial resistance genes and sequencing***

The genes encoding resistance to metronidazole (*nim*), MLS_B_ (*ermA, ermB, ermC*), tetracycline (*tetM, tetW*), and chloramphenicol (*catD*) were detected by PCR method using specific primers (Table 1) (11, 19, 20). The PCR products were separated and visualized, as mentioned above (Figure 1). The quinolone resistance-determining-regions (QRDRs) of *gyrA* and *gyrB* were also amplified, as described previously (Table 1) (21). The amplicons were sequenced by the ABI capillary system (Macrogen Research, Seoul, Korea). The sequences were aligned with the reference sequence of *C. difficile *630 (NCBI Reference Sequence: NC_009089.1), using online BLAST software (https://blast.ncbi.nlm.nih.gov/Blast.cgi).

***Statistical analysis***

The analysis was performed using SPSS^TM^ software, version 21.0 (IBM Co., Armonk, NY, USA). The distribution of MLS_B_ resistance genes among clindamycin non-susceptible and susceptible isolates was calculated by Chi-square and Fisher’s exact tests for each gene. The prevalence of tetracycline resistance genes among tetracycline non-susceptible and susceptible isolates was also calculated by the above mentioned tests. A *P*-value of ≤ 0.05 was considered as statistically significant.

## Results

Gram-positive bacilli, with subterminal endospores were observed in Gram-stained smears. Their yellow colonies had horse stable odor and chartreuse fluorescence under the UV light. Moreover, the *tpi* gene was found in 100% of the isolates and they were molecularly confirmed. All the isolates possessed at least one toxin associated gene. The genes encoding binary toxin were found in six (13.3%) isolates. The *cdtA* was present in five (11.1%) isolates and only one strain carried both *cdtA* and *cdtB* genes (2.2%). As shown in Table 2, predominant toxin profile was *tcdA*^+^,* tcdB*^+^,* cdtAˉ*, *cdtBˉ* (82.2%) followed by *tcdA*^+^,* tcdB*^+^,* cdtA*^+^, *cdtBˉ* (11.1%), *tcdAˉ*,* tcdB*^+^,* cdtAˉ*, *cdtBˉ* (4.5%), and *tcdA*^+^,* tcdB*^+^,* cdtA*^+^, *cdtB*^+^ (2.2%).

According to MIC results, resistance to tetracycline (MIC ≥16 µg/ml), clindamycin (MIC ≥8 µg/ml) and moxifloxacin (MIC ≥8 µg/ml) was observed in 30 (66.7%), 27 (60%) and 19 (42.2%) isolates, respectively (Table 3). None of the strains showed resistance to other antibiotics tested. The details of MIC results, MIC_50_ and MIC_90_ of the tested antibiotics against the isolates are shown in Table 3. 

The distribution of the *ermB* gene was 57.8% and the *ermA *and* ermC* genes were not detected amongst the isolates. The presence of *ermB* gene in clindamycin non-susceptible isolates was more than susceptible isolates, significantly (*P*<0.001). Also, the frequency of *tetM* and *tetW* genes was 62.2% and 13.3%, respectively. The *tetM* gene was more prevalent in tetracycline non-susceptible in comparison to susceptible isolates with a significant correlation (*P*<0.001). However, other resistance-related genes including *nim* and *catD* were not found in any of the isolates. Sequencing analysis of *gyrA* and *gyrB* revealed that of 19 moxifloxacin resistant strains, 15 isolates possessed (Thr82→Ile) substitution in GyrA, and four isolates had (Asp426→Asn) substitution in GyrB (Table 2). 

**Table 1:** Primers used in the study

**Gene**	**Function**	**Primer sequence (5'- 3')**	**Product size (bp)**	**Reference**
***tpi***	Triose phosphate isomerase (housekeeping)	F: AAAGAAGCTACTAAGGGTACAAA R: CATAATATTGGGTCTATTCCTAC	230	
***tcdA***	Toxin A	F: AGATTCCTATATTTACATGACAATAT R: GTATCAGGCATAAAGTAATATACTTT	369	
***tcdB***	Toxin B	F: GGAAAAGAGAATGGTTTTATTAA R: ATCTTTAGTTATAACTTTGACATCTTT	160	
***cdtA***	Binary toxin	F: TGAACCTGGAAAAGGTGATG R: AGGATTATTTACTGGACCATTTG	375	
***cdtB***	Binary toxin	F: CTTAATGCAAGTAAATACTGAG R: AACGGATCTCTTGCTTCAGTC	510	
***nim***	Metronidazole resistance	F: ATGTTCAGAGAAATGCGGCGTAAGCG R: GCTTCCTTGCCTGTCATGTGCTC	458	
***ermA***	MLS_B_ resistance	F: TATCTTATCGTTGAGAAGGGATT R: CTACACTTGGCTTAGGATGAAA	139	
***ermB***	MLS_B_ resistance	F: CTCAAAACTTTTTAACGAGTG R: CCTCCCGTTAAATAATAGATA	711	
***ermC***	MLS_B_ resistance	F: CTTGTTGATCACGATAATTTCC R: ATCTTTTAGCAAACCCGTATTC	190	
***tetM***	Tetracycline resistance	F: TGGAATTGATTTATCAACGG R: TTCCAACCATACAATCCTTG	1000	
***tetW***	Tetracycline resistance	F: CATCTCTGTGATTTTCAGCTTTTCTCTCCC R: AGTCTGTTCGGGATAAGCTCTCCGCCG	457	
***catD***	Chloramphenicol resistance	F: ATACAGCATGACCGTTAAAG R: ATGTGAAATCCGTCACATAC	500	
***gyrA***	Quinolone resistance (mutation)	F: AATGAGTGTTATAGCTGGACG R: TCTTTTAACGACTCATCAAAGTT	390	
***gyrB***	Quinolone resistance (mutation)	F: AGTTGATGAACTGGGGTCTT R: TCAAAATCTTCTCCAATACCA	390	

MLSB: macrolide–lincosamide–streptogramin B

**Table 2:** Toxin profiles and antimicrobial resistance patterns of *Clostridioides (Clostridium)* *difficile*

**No. of isolates**	**Underlying disease**	**Toxin profile** **(** ***tcdA, tcdB*** ** / CDT)**	**Resistance pattern**	**Resistance genes**	**GyrA/GyrB substitution**
1	Gastrointestinal diseases (15)	*tcdA, tcdB*	CD, TET	*ermB, tetM*	-
2		*tcdA, tcdB*	CD, MXF	*ermB*	GyrA (Thr82→Ile)
3		*tcdA, tcdB*	CD, TET, MXF	*ermB, tetM*	GyrA (Thr82→Ile)
4		*tcdA, tcdB */* cdtA*	CD, TET, MXF	*ermB, tetM*	GyrB (Asp426→Asn)
5		*tcdA, tcdB*	CD, MXF	-	GyrA (Thr82→Ile)
6		*tcdA, tcdB*	TET	*tetM*	-
7		*tcdA, tcdB*	CD, TET	*ermB, tetM*	-
8		*tcdA, tcdB*	TET, MXF	*tetW*	GyrA (Thr82→Ile)
9		*tcdA, tcdB*	CD, MXF	*ermB*	GyrA (Thr82→Ile)
10		*tcdA, tcdB*	TET, MXF	*tetM, tetW*	GyrA (Thr82→Ile)
11		*tcdA, tcdB*	-	-	-
12		*tcdA, tcdB */* cdtA*	TET	*tetM*	-
13		*tcdA, tcdB */* cdtA*	TET, MXF	*tetM*	GyrA (Thr82→Ile)
14		*tcdA, tcdB*	CD, MXF	*ermB*	GyrA (Thr82→Ile)
15		*tcdA, tcdB*	CD, TET, MXF	*ermB, tetM*	GyrA (Thr82→Ile)
16	Hematologic disorders (10)	*tcdA, tcdB */* cdtA*	CD	-	-
17		*tcdA, tcdB*	CD	*ermB*	-
18		*tcdA, tcdB*	CD, TET	*ermB, tetM, tetW*	-
19		*tcdA, tcdB*	CD, TET, MXF	*ermB, tetM*	GyrB (Asp426→Asn)
20		*tcdA, tcdB*	CD, TET	*ermB, tetM*	-
21		*tcdA, tcdB*	CD, TET	*ermB, tetM*	-
22		*tcdA, tcdB*	CD	*ermB*	-
23		*tcdA, tcdB*	CD, TET	*ermB, tetM*	-
24		*tcdA, tcdB*	TET, MXF	*ermB, tetM*	GyrA (Thr82→Ile)
25		*tcdA, tcdB*	CD, TET, MXF	*ermB, tetM*	GyrA (Thr82→Ile)
26	Liver diseases (6)	*tcdA, tcdB*	TET	*tetM, tetW*	-
27		*tcdA, tcdB*	CD	*ermB*	-
28		*tcdA, tcdB */* cdtA*	CD, TET	*ermB, tetM*	-
29		*tcdA, tcdB,*	TET	*ermB, tetM*	-
30		*tcdA, tcdB*	TET, MXF	*tetM*	GyrA (Thr82→Ile)
31		*tcdA, tcdB */* cdtA, cdtB*	CD, TET, MXF	*ermB, tetM*	GyrA (Thr82→Ile)
32	Kidney diseases (5)	*tcdA, tcdB*	CD, MXF	*ermB*	GyrB (Asp426→Asn)
33		*tcdA, tcdB*	CD, TET	*tetM*	-
34		*tcdB*	CD, TET	*tetM*	-
35		*tcdA, tcdB*	TET, MXF	*tetM*	GyrA (Thr82→Ile)
36		*tcdA, tcdB*	TET	*ermB, tetM, tetW*	-
37	Metabolic disorders (4)	*tcdA, tcdB*	-	-	-
38		*tcdA, tcdB*	-	-	-
39		*tcdA, tcdB*	TET	*tetW*	-
40		*tcdB*	CD, TET	*ermB, tetM*	-
41	Pneumonia (2)	*tcdA, tcdB*	MXF	*-*	GyrA (Thr82→Ile)
42		*tcdA, tcdB*	CD, MXF	*ermB*	GyrB (Asp426→Asn)
43	Eye diseases (2)	*tcdA, tcdB*	TET	*tetM*	-
44		*tcdA, tcdB*	-	-	-
45	Osteosarcoma (1)	*tcdA, tcdB*	CD, TET	*ermB, tetM*	-

CD: Clindamycin; TET: Tetracycline; MXF: Moxifloxacin

**Table 3:** Minimum inhibitory concentrations (MICs) of the tested antibiotics against *Clostridioides (Clostridium) difficile* isolates

**Antibiotic**	**MIC (µg/mL)**												
	0.125	0.19	0.25	0.38	0.5	0.75	1	1.5	2	4	8	16	≥32
**Vancomycin**	4	10	8	9	3	**7**	4						
**Metronidazole**					12	16	7	5	**5**				
**Clindamycin**							3		12	3	4	2	**21**
**Tetracycline**					2		5		4	2	2	11	**19**
**Moxifloxacin**					3		5		14	4	5	7	**7**
**Chloramphenicol**					8		15		11	6	**2**	3	

MIC: Minimum inhibitory concentration; underlined: MIC_50_; boldface: MIC_90_

## Discussion

In the present study, toxin profiles and antimicrobial susceptibilities of CDI causative isolates were investigated. All the isolates were toxigenic and carried at least one toxin associated gene (Table 2). Similarly, a high distribution of the genes encoding toxins A and B amongst clinical *C. difficile* isolates was reported in various studies (1, 5, 22-24). The main role of these virulence factors to cause CDI had been well characterized (3, 4).

Our findings indicated that one (2.2%) isolate had both CDT related genes (*cdtA* and *cdtB*) and five (11.1%) isolates harboured *cdtA* gene without *cdtB* (Table 2). Clinical *C. difficile* isolates with only one of the two components gene (*cdtA* or *cdtB*) have been reported from other Asian counties (9, 25). It seems that this pattern of binary toxin genes is prevalent in our geographical region. Similar to our findings, in the other study from Iran, these two genes were detected in a CDT positive isolate simultaneously (26). Presence and expression of binary toxin genes create synergy with other toxins and increase the pathogenicity of *C. difficile *(25, 27).

According to our results, the most frequent toxin profile was A^+^ B^+ ^CDT ¯ . Although a different pattern was published as the major genotype from Kerman, Iran in 2017 (26), but A^+^ B^+ ^CDT ¯ profile was predominant in the several studies around the world (5, 28-32).

MICs results showed that all the isolates were completely inhibited by vancomycin and metronidazole (Table 3). Various studies showed the same results and these agents were effective against their isolates (1, 5, 9, 24, 33-37). Although vancomycin and metronidazole were efficient choices against CDI in our study, however, their prescription should be under control for prevention of the emergence of resistant strains. In recent years, vancomycin and/or metronidazole resistance has been observed in Iran and the other countries (8, 32, 38, 39). These data suggest that CDI therapy by the mentioned antibiotics could be problematic in the future. In the current study, chloramphenicol resistance and its related gene (*catD*) were not found amongst the isolates (MICs ≤ 16) (Table 3). In contrast, resistance to this antibiotic was described in previous studies (11, 32, 35, 40, 41). The rate of chloramphenicol prescription in our investigated hospitals is very low. Therefore, it can justify lack of chloramphenicol resistance.

We found that 30 (66.7%) *C. difficile* isolates displayed tetracycline resistance (MIC ≥16) (Table 3). They carried at least one *tetM *or* tetW* gene and four strains had both genes (Table 2). Resistance to tetracycline was shown in several studies (10, 20, 31, 35, 37, 38). Our findings were in line with the previous report that showed that the predominant *tet* gene in *C. difficile* was* tetM* and the second frequent one was *tetW *(20). However, in a study conducted by Abuderman *et al*., only *tetM* was observed amongst the resistant *C. difficile* isolates (9). One of the reasons for notable rates of tetracycline resistance and *tet* genes distribution is the presence of *tet* cluster of genes on transferable mobile elements (7, 8). 

The percentage of the isolates resistant to clindamycin (MIC ≥8) and moxifloxacin (MIC ≥8) was 60% (27/45) and 42.2% (19/45), respectively. The *ermB* gene was found in 26 (57.8%) isolates and *ermA *and* ermC* genes were not detected amongst any of the strains. As mentioned in several studies, *ermB* had a key role in MLS_B_ resistance of* C. difficile* isolates (9-11, 20, 42). According to our findings, some *ermB*-negative strains were clindamycin resistant (Table 2). Other mechanisms such as efflux pumps or *cfr* gene may be responsible for resistance. The *cfr* gene, which encodes an RNA methyltransferase, can confer MLS_B_ resistance in *erm*-negative bacteria (8). On the other hand, three susceptible isolates carried the *ermB *gene. This sensitivity is probably related to the insufficient expression of the gene.

Sequencing analysis revealed that all moxifloxacin resistant isolates possessed substitution in GyrA or GyrB (Table 2). The substitution Thr82 to Ile in GyrA was found in the majority of the resistant strains (15/19). Furthermore, substitution Asp426 to Asn in GyrB also accounted for resistance to other moxifloxacin resistant isolates (4/19). The same substitution in GyrA was remarkable amino acid changes in previous studies (21, 43). 

## Conclusion

The most frequent toxin profile was *tcdA*^+^,* tcdB*^+^,* cdtAˉ*, *cdtBˉ* (82.2%), and only one isolate harboured all toxin associated genes (*tcdA*^+^,* tcdB*^+^,* cdtA*^+^, *cdtB*^+^) (2.2%). The genes encoding binary toxin were also found in six (13.3%) strains. Only one strain carried both *cdtA* and *cdtB* genes (2.2%). Resistance to tetracycline, clindamycin and moxifloxacin was observed in 30 (66.7%), 27 (60%) and 19 (42.2%) isolates, respectively. Metronidazole, vancomycin and chloramphenicol resistance was not seen amongst the isolates. The distribution of the *ermB* gene was 57.8% and the *ermA *and* ermC* genes were not detected. The *tetM* and *tetW* genes were found in 62.2% and 13.3%, respectively. Other resistance-related genes including *nim* and *catD* were not observed in any of the isolates. Sequencing analysis of *gyrA* and *gyrB* revealed that the substitution of Thr82 to Ile in GyrA was the major amino acid change in the resistant strains (15/19). Also, substitution of Asp426 to Asn in GyrB was responsible for resistance to other moxifloxacin resistant isolates (4/19). Our data indicated notable virulence and antibiotic resistance traits amongst the isolates. Therefore, infection control strategies should be performed in order to curb the colonization and dissemination of *C. difficile* strains in hospital.
